# What is the preferred timing for surgery after proximal humerus fractures: an e-modified Delphi Survey Study

**DOI:** 10.1016/j.xrrt.2026.100865

**Published:** 2026-08-22

**Authors:** Stijn R.J. Mennes, Huub H. de Klerk, Reinier W.A. Spek, Neal C. Chen, Ross K. Leighton, Bhavin Jadav, Ruurd L. Jaarsma, Job N. Doornberg, Michel P.J. van den Bekerom, Timing for Shoulder Surgery Consortium (TSSC), T.D.W. Alta, G.I. Bain, H. van der Bracht, B.A. ten Brinke, D.F.P. van Deurzen, M.D. Derias, E.T. Ek, D.F. Ferguson, T. Gosens, J.C. Goslings, S.G. Greiner, S. Grewal, P.G. Guy, D.H. den Hartog, T.J.A. Karelse, Y. Kleinlugtenbelt, L.M. Kok, R. Lohre, O. van der Meijden, P. Moroder, S.J.B. Nijs, A. van Noort, M.K. Patel, D. Petré, E.E.J. Raven, C.R. Rosso, M.J.S. Sandow, M.N. van Sterkenburg, A. van Tongel, B.A. Twigt, E.J.D. Veen, H.C. van der Veen, C.P.J. Visser, R. Wessel, J.W. White

**Affiliations:** a Department of Orthopaedics and Trauma Surgery, Flinders Medical Centre and Flinders University, Adelaide, Australia; b Shoulder and Elbow Unit, Department of Orthopaedic Surgery, OLVG, Amsterdam, the Netherlands; c Faculty of Behavioural and Movement Sciences, Department of Human Movement Sciences, Vrije Universiteit Amsterdam, Amsterdam Movement Sciences, Amsterdam, the Netherlands; d Hand and Arm Research Collaborative, Department of Orthopaedic Surgery, Massachussets General Hospital, Harvard Medical School, MA, USA; e Department of Orthopaedic Surgery, University Medical Centre Groningen, Groningen, the Netherlands; f Harvard Medical School, Boston, MA, USA; g Division of Orthopaedic Surgery, Dalhousie University, Halifax, NS, Canada

**Keywords:** Delphi, Consensus, Timing, Surgery, Proximal humerus fractures, ORIF, Arthroplasty

## Abstract

**Background:**

Certain clinically selected proximal humerus fractures (PHFs) require operative treatment to preserve shoulder function. There is no consensus on the timing of surgery after PHFs, and guidelines are lacking. This study utilized the Delphi method to (1) find consensus on the preferred timing for multiple PHF patterns potentially requiring operative treatment and (2) assess statistical stability of consensus.

**Methods:**

A three-round electronic modified Delphi study was conducted between May and October 2025. Sixty-one shoulder experts from Australia, Europe, Asia, and North America participated. The expert panel assessed preferred time to surgery for PHF patterns potentially requiring surgery (either open reduction and internal fixation or arthroplasty) according to literature. The established timeframes were (a) within 2 days, (b) 3-5 days, (c) 6-14 days, or (d) after 14 days of initial trauma. Consensus was predefined as ≥80% agreement among experts and stable consensus as ≥80% agreement in both second and third rounds.

**Results:**

For open reduction and internal fixation, consensus was observed to treat PHFs with ≥35% intra-articular involvement within 3-5 days, and plexopathy with distal motor and/or sensory symptoms within 2 days. Stable consensus was reached to treat dislocation of the humeral head to the axilla and vascular compromise with adequate distal perfusion within 2 days. Closed vascular injury with active bleeding in hemodynamically unstable patients or distal vascular deficits should be treated immediately but could wait up to 2 days in hemodynamically stable patients with adequate distal perfusion. For arthroplasty, consensus was achieved to treat PHFs with varus malalignment of the humeral head or (almost) complete translation of the shaft within 6-14 days. Stable consensus was observed to perform arthroplasty within 2 days for PHFs with dislocation of the humeral head to the axilla with plexopathy or vascular compromise with adequate distal perfusion within 2 days.

**Conclusion:**

This study provides insights on consensus among international shoulder surgeons for preferred timing of surgery for PHFs for urgent indications. Importantly, consensus was not achieved for most non-emergent PHFs, underscoring the area of uncertainty in clinical practice regarding the optimal treatment window. This study highlights the absence of agreement and lack of high-quality evidence to guide physicians on the timing of operative treatment for PHFs and can be a starting point for further clinical research and developing guidelines on the timing of surgical interventions for PHFs.

Proximal humerus fractures (PHFs) are observed frequently, encompassing 5%-6% of all fractures.^5^ The majority of PHFs is non-displaced to minimally displaced and can be managed non-operatively.^16^^,^^21^ However, some require operative treatment in selected patients due to certain fracture characteristics associated with poor outcomes, such as comminution, intra-articular involvement (former head-split fractures), (almost) complete head-shaft translation, and severe varus angulation.^6^^,^^10^^,^^12^

Controversy exists whether these patients should receive operative treatment in the acute setting.^20^ Moreover, there is no consensus on the timing of acute operative interventions. Delaying open reduction and internal fixation (ORIF) more than 5 days may be associated with increased complications, such as malreduction, loss of fixation, intra-articular screw protrusion, and avascular necrosis.^26^ On the other hand, optimizing patients before shoulder surgery (eg, medical optimization, thromboembolism risk management, or peri-operative surveillance) may prevent adverse events and unplanned readmissions.^14^ For femoral neck fractures, guidelines exist regarding the timing of surgery.^19^ However, similar guidelines for PHFs have yet to be established. The urgency of intervention may be guided by specific fracture characteristics. For example, delaying surgery more than 2 days after head-split and proximal humerus dislocation fractures may increase the risk of avascular necrosis,^23^^,^^26^ whereas evidence for surgical timing for other fracture patterns remains scarce. Furthermore, clinical comparisons of acute versus delayed surgery may be misleading, as patients in the latter group are not representative for the average person with a similar fracture.^22^

To address this lack of guidance and uncertainty in evidence, collecting opinions from experienced international shoulder surgeons could provide unity in decision-making on the timing of surgical interventions after PHFs. Delphi studies are commonly undertaken to seek expert opinion in a structured method and find consensus on a specific topic.^7^^,^^18^ A Delphi process can help steer future research, as consensus can guide subsequent studies to establish clinical evidence for guidelines, whereas disagreement identifies areas warranting additional research. Given the variety in surgical decision-making regarding PHFs across countries,^11^ and likely across continents, it is beneficial to involve experts from multiple continents.

Therefore, this study aimed to collect expert opinions from an intercontinental panel of experienced shoulder surgeons utilizing the Delphi method to (1) find consensus considering the optimal timing for multiple PHF patterns that require operative treatment according to literature and (2) assess statistical stability of consensus, may consensus exist.

## Materials and methods

This study is an e-modified Delphi study consisting of 3 rounds of surveys between May 2025 and October 2025. This study was conducted according to the ACcurate COnsensus Reporting Document guidelines, Checklist for Reporting Results of Internet E-Surveys and Checklist for Reporting of Survey Studies guidelines.^8^^,^^9^^,^^25^ The study protocol was not prospectively registered.

### Recruitment of the expert panel

The authors (N.C., R.L., J.D., M.B., and R.J.), experienced orthopedic surgeons and researchers, provided a list of internationally recognized shoulder surgeons from their continents (Australia, Europe, and North America). Potential panelists were identified based on their current clinical practice in shoulder surgery and/or orthopedic trauma, their routine involvement in the management of PHFs, and their established clinical and/or academic involvement in this field. They were approached for participation through a personal e-mail with information concerning the study. Participation was voluntary and no incentive besides group authorship for this study was offered. Panelists who confirmed their participation were asked to report their years of practice, experience, country of surgical training, and country of current practice. All participants were required to confirm that they actively treated patients with PHFs and that they were board certified surgeons. Shoulder surgeons who missed the first survey round but were willing to participate were still eligible to fill out subsequent surveys.

### Questionnaire

The questionnaires of this e-modified Delphi study were created and provided to the panel using Castor EDC (Amsterdam, The Netherlands), an electronic data capture system.^4^ This data capture system allows for automatic data collection. One of the researchers (S.M.) was the primary contact and coordinated the distribution of the questionnaires. S.M. also developed subsequent surveys based on the panel's responses. Every round was tested 3 times before distribution to the expert panel by 2 researchers (S.M., H.K.). Test rounds were timed, and an estimate of the time required to complete the survey was added to the survey invitation.

According to Delphi methodology,^18^ all answers from panelists to the survey rounds were anonymous. All questions in the survey were obligatory before panelists were able to submit the questionnaire. Every participant received 1, unique link that could be filled out once to prevent multiple entries. Beforehand, the authors determined that the Delphi process would be terminated after three rounds, and no additional stopping criteria were applied.

### First round survey

The first round aimed to introduce the proposed PHF patterns to the expert panel. The list of patterns was developed from a targeted literature review up to March 2025. Landmark papers, systematic reviews, treatment algorithms, and clinical studies were considered. Moreover, input from study team experts was also implemented. Before distributing the first survey, all authors reviewed the list. A meeting was scheduled with the study team to review the fracture patterns and to engage in discussion in case of disagreement on the description or inclusion of a case. The panelists were asked to provide feedback on the list regarding missed cases and possible confusion. The fracture patterns were divided in an ‘ORIF’ and ‘Arthroplasty’ group. The fracture patterns included in the ORIF and arthroplasty groups were selected based on a literature review and the clinical expertise of the study team. A fracture pattern was included in a group when the corresponding procedure was considered a clinically plausible treatment option. The 2 groups were not mutually exclusive, as several fracture patterns may be treated with either ORIF or arthroplasty depending on patient characteristics, bone quality, reconstructive potential, and surgeon preference. The study did not assess the choice between ORIF and arthroplasty; panelists were asked to indicate their preferred timing of surgery assuming that the decision for operative treatment and the choice of procedure had already been made.

The ORIF group consisted of the following fracture patterns: PHF without characteristics, PHF with dislocation of the humeral head to the axilla, PHF with “locked” posterior fracture-dislocation of the humeral head, PHF with ≥35% intra-articular involvement, (almost) complete translation of shaft (<5 mm overlap), varus malalignment of humeral head (neck-shaft angle [NSA] ≤100°), tuberosity (greater and/or lesser) displacement >5 mm, PHF with plexopathy with distal motor and/or sensory symptoms, PHF with vascular injury and active bleeding, and PHF with vascular compromise and adequate distal perfusion. Based on the input from the panelists, an unstable medial hinge and valgus malalignment of humeral head (NSA ≥150°) were added to the list. The arthroplasty group consisted of the following: PHF without dislocation of humeral head to axilla or neurovascular injury, PHF with dislocation of humeral head to axilla without plexopathy, PHF with dislocation of the humeral head to the axilla and plexopathy, PHF with axillary nerve injury and compromised deltoid function, PHF with vascular compromise and adequate distal perfusion, PHF with “locked” posterior fracture-dislocation of the humeral head, PHF with ≥35% intra-articular involvement, varus malalignment of the humeral head (NSA ≤100°), and (almost) complete translation of shaft (<5% overlap). Fracture patterns that were included in the survey are also listed in Supplementary File 1.

### Second round survey

The second round aimed to identify the timing of surgery after the PHF patterns possibly requiring surgery according to the literature with additions by the expert panel from the first round. Participants were asked to provide a timeframe (in days), within which they would surgically manage a patient for each specific injury including optional rationale, given that the choice for surgery was already made. The following timeframes were selected based on a literature review^2^^,^^17^^,^^19^^,^^23^^,^^24^^,^^26^ and clinical expertise: (a) within 2 days, (b) within 3-5 days, (c) within six to fourteen days, or (d) after fourteen days of the initial trauma.

### Third round survey

**Figure 1:**
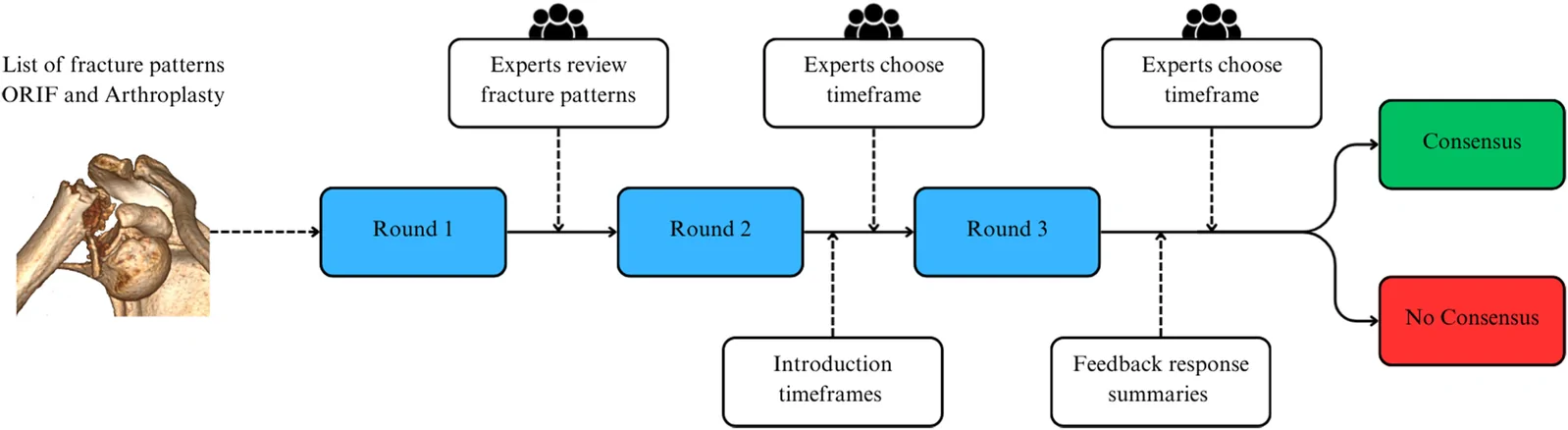
Schematic overview of the three-round Delphi process.

In the third and final round, panelists received the same questionnaire as in the second round with controlled feedback, including a summary of the results of the second round that emerged from statistical analysis of the anonymous responses and the provided rationales. This allowed participants to adjust their initial answers if they wanted to (Fig. 1, surveys are available as Supplementary Files 1, 2, and 3).

### Statistical analysis

Frequencies were calculated for each fracture pattern to provide them to the panel in the second-round survey. Early terminated questionnaires were analyzed using a per-question complete-case analysis approach. Consensus was predefined as ≥ 80% agreement (80-89%), strong consensus as ≥ 90% (90-99%), and unanimous consensus as 100%.^7^ Agreement ≥80% in both the second and third rounds were considered as stable consensus.^18^ Only answers from participants who participated in both rounds were included for stable consensus analysis. Data analysis was performed with R (R Foundation for Statistical Computing, Vienna, Austria).

## Results

### Panelists

**Figure 2:**
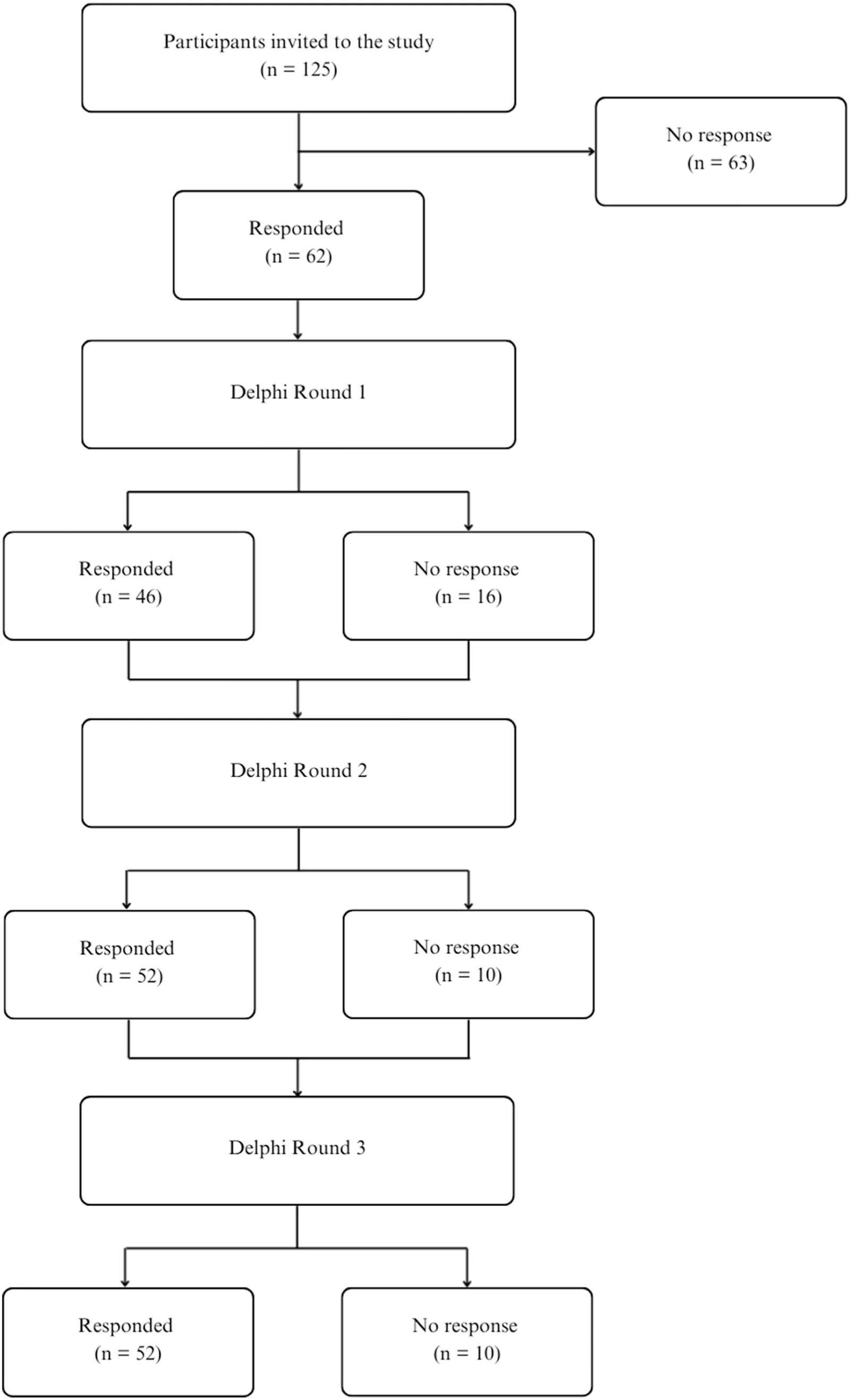
Flowchart of total respondents and respondents for each specific round.

Sixty-two of the 125 (50%) invited potential panelists were willing to participate and confirmed they had sufficient experience and expertise to comment on the timing of surgery after PHFs (Fig. 2). Forty-six panelists (74%) completed the first round, 50 (81%) completed the second round, and 52 (84%) completed the third round. Forty-seven panelists (76%) completed both the second and third round and were included in the stability analysis. The respondents received surgical training in Australia (13%), Europe (74%), Asia (4%), or North America (9%). Currently, 10 (22%) surgeons are practicing in Australia, 31 (67%) in Europe, 1 (2%) in Asia, and 4 (9%) in North America. The majority has more than 10 years of experience (70%), and more than half of the respondents operatively treat over 20 PHFs per year (61%).

### Timing of surgery after proximal humerus fractures treated with open reduction and internal fixation

Out of the proposed fracture patterns treated with ORIF, stable consensus on the preferred surgical timing was reached to treat PHFs with dislocation of the humeral head to the axilla and vascular compromise with adequate distal perfusion within 2 days. Vascular injury with active bleeding in hemodynamically unstable patients or distal vascular deficits should be treated immediately in unstable patients but can be treated within 2 days in stable patients with adequate distal perfusion as per the consensus. Consensus existed to treat PHFs with plexopathy and distal motor and/or sensory symptoms within 2 days, and ≥35% intra-articular involvement within 3-5 days, although this was not a stable consensus (Table 1).

**Table I:** Fracture types treated with open reduction and internal fixation.

Fracture type	Preferred timeframe	Agreement, %	Consensus	Stable consensus‡
PHF without characteristics∗	Within 6-14 d	64		
PHF with dislocation of the humeral head to the axilla	Within 2 d	94	Strong consensus	Yes (85%)
PHF with “locked” posterior fracture-dislocation of the humeral head†	Within 2 d	75		
PHF with ≥35% intra-articular involvement	Within 3-5 d	81	Consensus	No (53%)
(Almost) complete translation of shaft (<5 mm overlap)	Within 3-5 d	69		
Varus malalignment of humeral head (NSA ≤100°)	Within 6-14 d	60		
Valgus malalignment of humeral head (NSA ≥150°)	Within 6-14 d	60		
Tuberosity (greater and/or lesser) displacement >5 mm	Within 6-14 d	71		
Unstable disruption of the medial hinge	Within 3-5 d	56		
PHF with plexopathy with distal motor and/or sensory symptoms	Within 2 d	96	Strong consensus	No (79%)
PHF with vascular injury and active bleeding	Within 2 d	100	Unanimous consensus	Yes (100%)
PHF with vascular compromise and adequate distal perfusion	Within 2 d	100	Unanimous consensus	Yes (93.6%)

*PHF*, proximal humerus fracture; *NSA*, neck-shaft angle.

∗ Dislocation, intra-articular involvement, complete shaft translation, varus- or valgus malalignment, tuberosity displacement, unstable medial hinge, or neurovascular injury.

† Head fixated behind posterior glenoid with reversed Hill-Sachs lesion.

‡ Stable consensus reflects agreement ≥80% in both second and third rounds, reported with the percentage from round 2.

### Timing of surgery after proximal humerus fractures treated with arthroplasty

For fracture patterns treated with arthroplasty, stable consensus was reached in 2 cases. Panelists agreed that PHFs with dislocation of the humeral head to the axilla and plexopathy and vascular compromise with adequate distal perfusion should be operated on within 2 days. For varus malalignment of the humeral head (NSA ≤100°) and PHFs with (almost) complete translation of the shaft, consensus existed to treat within 6-14 days. However, consensus for these patterns was not stable (Table 2).

**Table II:** Fracture types treated with arthroplasty.

Fracture type	Preferred timeframe	Agreement, %	Consensus	Stable consensus‡
PHF without dislocation of humeral head to axilla or neurovascular injury	Within 6-14 d	79		
PHF with dislocation of humeral head to axilla without plexopathy	Within 3-5 d	64		
PHF with dislocation of the humeral head to the axilla and plexopathy∗	Within 2 d	98	Strong consensus	Yes (83%)
PHF with axillary nerve injury and compromised deltoid function	Within 2 d	62		
PHF with vascular compromise and adequate distal perfusion	Within 2 d	100	Unanimous consensus	Yes (85%)
PHF with “locked” posterior fracture-dislocation of the humeral head†	Within 3-5 d	60		
PHF with ≥35% intra-articular involvement	Within 6-14 d	77		
Varus malalignment of the humeral head (NSA ≤100°)	Within 6-14 d	81	Consensus	No (62%)
(Almost) complete translation of shaft (<5% overlap)	Within 6-14 d	81	Consensus	No (62%)

*PHF*, proximal humerus fracture; *NSA*, neck-shaft angle.

∗ Distal motor and/or sensory symptoms.

† Head fixated behind posterior glenoid with reversed Hill-Sachs lesion.

‡ Stable consensus reflects agreement ≥80% in both second and third rounds, reported with the percentage from round 2.

## Discussion

This Delphi study reached consensus regarding timing of surgical intervention for 5 proximal humeral fracture cases treated with ORIF and 4 cases treated with arthroplasty. For ORIF, stable consensus (≥80% agreement in both second and third rounds) was observed to treat PHFs with dislocation of the humeral head to the axilla, vascular injury with active bleeding (might require more urgent treatment), and vascular compromise with adequate distal perfusion within 2 days. In the arthroplasty group, stable consensus was reached to treat PHFs with dislocation of the humeral head to the axilla and plexopathy and vascular compromise with adequate distal perfusion within 2 days.

Several limitations should be considered when interpreting our results. First, experts were asked to choose a timeframe in an idealized situation. In actual practice, 1 needs to deal with multiple factors, like availability of operating staff and rooms and the patients' comorbidities, which could potentially result in operative delay. Second, not all continents were equally represented in this study. Surgeons from other continents may differ from the ones participating in our study because of differences in treatment convictions and scientific literature (eg, Spanish, French, or Chinese language), leading to potential bias in our results. Third, we aimed to include all fracture patterns possibly requiring surgery. Nevertheless, some surgeons would not operate on some fracture types included in our study as treatment variability exists across continents and even countries, possibly leading to a lower participation rate or easier conformation to the majority's opinion. Fourth, the chosen timeframes for this study may seem arbitrary. Consequently, consensus might suggest that the acute fracture patterns consensus was reached for may be operated within two days. Importantly, it should be noted that some acute operation indications, such as vascular injury with active bleeding, need immediate operative intervention. Thus, the time-window for surgery is shorter than the 2-day timeframe when considering the patient's life and limb. However, these timeframes were not arbitrary: we proposed timeframes previously established by others in multiple prior clinical studies.^2^^,^^17^^,^^19^^,^^23^^,^^24^^,^^26^ While further evaluating timeframes within the two day interval (eg, 6, 12, or 24 hours) may have provided additional insights, these were not incorporated to minimize respondent burden and maximize participation and completion of our surveys.

### Timing of surgery after proximal humerus fractures treated with open reduction and internal fixation

Currently, there is little evidence to provide guidance in the timing of surgery in PHFs. Guidelines on the time to surgery do not exist and studies have reported contrasting results. In this study, all experts preferred to treat the included fracture patterns with ORIF within 14 days. A sub-analysis from a randomized controlled trial on PHFs did not show differences in functional outcomes in patients with PHFs and delayed operative treatment after 8 days or more, compared with patients treated within 8 days.^19^ Contrary to their findings, other studies reported unfavorable outcomes when operative treatment was delayed. Menendez et al^17^ reported increased inpatient morbidity, post-operative length of stay, and non-routine discharge rates in patients receiving surgery more than 3 days after admission. Moreover, ORIF for PHFs after 5 days is associated with increased complications.^26^ While avascular necrosis may not be related to time to surgery for PHFs,^1^ delayed surgery after 2 days in proximal humeral dislocation fractures may be significantly associated with higher avascular necrosis rates as the scarce blood supply is often disrupted.^23^^,^^26^ In accordance with these findings, the consensus was to perform ORIF for fracture dislocations within the 2-day timeframe. Siebenburger et al,^26^ recommended to treat intra-articular fractures within 2 days of trauma. However, experts in this study had a consensus recommendation to treat intra-articular fractures without glenohumeral dislocation within 3-5 days. There was, however, a 30% difference between the second and third round, indicating some uncertainty.

### Timing of surgery after proximal humerus fractures treated with arthroplasty

Although no distinction in fracture patterns was made, a 2023 review reported superior functional outcomes, less complications, and lower risk of revision surgery when reverse arthroplasty for fractures is performed within four weeks of injury.^15^ This may be due to better tuberosity healing after acute surgery, leading to superior range of motion and functional outcomes.^13^^,^^24^ Delayed operative treatment may lead to development of callus, migration, or malunion of the greater tuberosity and soft-tissue scarring, complicating reduction and repair when reverse arthroplasty is performed.^15^^,^^24^ Moreover, Boileau et al^3^ suggest that a greater tuberosity osteotomy is the greatest predictor for poor outcomes of arthroplasty. In line with these findings, almost all experts in this study preferred to perform arthroplasty within 2 weeks for the proposed fracture patterns.

Our study evaluated expert opinion on the preferred time to surgery for proximal humeral fracture patterns possibly requiring surgery. In general, there was consensus for surgical indications, requiring immediate intervention to prevent devastating outcomes. Patients with a PHF, vascular injury, and active bleeding should be operated as soon as possible, while vascular compromise with intact vascularization and patients with plexopathy require an operation within two days. For other PHF patterns, such as intra-articular involvement, varus or valgus malalignment, shaft translation, and tuberosity displacement, timing of surgery may rely more on personal surgical preferences. A recent Delphi study on the treatment choice for PHFs concluded that there is limited consensus on multiple PHF patterns.^27^ Disagreement was also observed in our study when assessing preferred timing of surgical intervention for the fracture patterns deemed appropriate for non-urgent treatment. This may reflect the contrasting clinical outcomes, and many studies do not distinguish between individual sub-types. Consequently, clinical evidence may be too heterogeneous to draw conclusions for sub-groups of PHFs. This emphasizes the need for more studies on the relation between time to surgery and clinical outcomes of surgery for these patterns, while considering the timeframes and achieved consensus in this study.

## Conclusion

Consensus was only reached on the preferred timing of surgery for multiple proximal humeral fracture patterns treated with ORIF or arthroplasty requiring urgent intervention. Consensus was not achieved for the majority of non-emergent PHF patterns, underscoring the area of uncertainty in clinical practice regarding the optimal treatment window. The findings of this study highlight the absence of agreement and lack of high-quality evidence to guide physicians on the timing of operative treatment for PHFs. This study can serve as a starting point for further clinical research and development of clinical guidelines in the endeavor to unity in timing of surgical interventions after PHFs.

## Acknowledgments

The Timing for Shoulder Surgery Consortium study group is as follows: T.D.W. Alta, G.I. Bain, H. van der Bracht, B.A. ten Brinke, D.F.P. van Deurzen, M.D. Derias, E.T. Ek, D.F. Ferguson, T. Gosens, J.C. Goslings, S.G. Greiner, S. Grewal, P.G. Guy, D.H. den Hartog, T.J.A. Karelse, Y. Kleinlugtenbelt, L.M. Kok, R. Lohre, O. van der Meijden, P. Moroder, S.J.B. Nijs, A. van Noort, M.K. Patel, D. Petré, E.E.J. Raven, C.R. Rosso, M.J.S. Sandow, M.N. van Sterkenburg, A. van Tongel, B.A. Twigt, E.J.D. Veen, H.C. van der Veen, C.P.J. Visser, R. Wessel, and J.W. White.

## Disclaimers:

Funding: No funding was disclosed by the authors.

Conflicts of interest: Stijn R.J. Mennes received personal funding of $0—10,000 from Michael van Vloten Foundation (Rotterdam, the Netherlands), $10,000 from C&W de Boer Stichting (Groningen, the Netherlands), and $0—10,000 from Cultuurfonds (Amsterdam, the Netherlands) and USC Scholarship Foundation (Utrecht, the Netherlands), all unrelated to this study. OLVG receives fellowship support from Smith & Nephew, unrelated to this study. Any additional authors, their immediate families, and any research foundations with which they are affiliated have not received any financial payments or other benefits from any commercial entity related to the subject of this article.

## Supplementary Data

Delphi Timing of Surgery After PHFs Round 1

Delphi Timing of Surgery After PHFs Round 2

Delphi Timing of Surgery After PHFs Round 3
