# Supplementary material for: What is the preferred timing for surgery after proximal humerus fractures: an e-modified Delphi Survey Study
Source: JSES Rev Rep Tech. 2026 Aug 22;6(4):100865. doi: 10.1016/j.xrrt.2026.100865 (PMC13599625; doi:10.1016/j.xrrt.2026.100865)
Supplement: Supplementary file 1 [file mmc1.pdf]

Survey Package 'Delphi Timing of Surgery After PHFs - Round 1'

Delphi Timing of Surgery After PHFs - Round 1 - Introduction

Dear panellist,

Thank you for participating in this Delphi consensus project. This online survey is the first of three rounds of this e-modified Delphi study. First, we will ask you about your level of experience and affiliations. Subsequently, you will be shown a list of specific proximal humerus fracture types. These cases have been carefully selected based on a literature review (up to July 2024) and clinical experience. Please note that this study focuses on **the optimal timing of surgical management**, not the type of management.

We ask you to review these fracture types assuming you have decided to treat them operatively. You will have the opportunity to comment on the list and add any additional fracture types. Please make sure that you answer all the questions and complete this survey. You may leave the survey and return to complete it at any time. You may go back and review your previous answers. Once you complete the survey, it will be locked and your answers will be definitive.

Survey 'Surgeon demographics and affiliations'

Surgeon demographics and affiliations - Introduction

First, we would like to gain insights into your level of experience and affiliations. Please fill out the following questions.

Surgeon demographics and affiliations - Demographics and affiliations

Number	Question	Answers
1.1	How many years of surgical experience do you have in orthopaedic trauma?	◯ 1 - 5 years<br>◯ 6 - 10 years<br>◯ 11 - 20 years<br>◯ > 20 years
1.2	Approximately, how many proximal humerus fractures do you treat annually?	◯ 1 - 20<br>◯ 21 - 50<br>◯ 51 - 100<br>◯ >100

1.3	On which continent did you receive your surgical training?	◯ Africa<br>◯ Asia<br>◯ Europe<br>◯ North America<br>◯ Oceania<br>◯ South America
1.4	On which continent do you practice medicine?	◯ Africa<br>◯ Asia<br>◯ Europe<br>◯ North America<br>◯ Oceania<br>◯ South America
1.5	What is your department?	☐ Orthopaedic Surgery<br>☐ Trauma Surgery<br>☐ Other
1.5.1	***If 'What is your department?' is equal to 'Other' answer this question:***<br>Please state your department	

Survey 'Personal information'

Personal information - Introduction

All information will remain confidential and all answers are anonymous. Your personal information will solely be used to contact you for subsequent survey rounds, to analyse the stability of the Delphi process and to acknowledge you as a group author if you complete the three rounds of this study.

Personal information - Personal information

Number	Question	Answers
	All information will remain confidential and all answers are anonymous.	
1.1	Please state your first name.	

1.2	Please state your initials.	
1.3	Please state your surname.	
1.4	Do you have any conflicts of interest to declare	◯ Yes<br>◯ No
1.4.1	***If 'Do you have any conflicts of interest to declare' is equal to 'Yes' answer this question:***<br>Please state your conflict of interest.	

Survey 'List of proximal humerus fractures - ORIF'

List of proximal humerus fractures - ORIF - Introduction

In the following part, we present a list of specific proximal humerus fractures. Please note that this study focuses on **the optimal timing of surgical management**, not the type of management. We ask you to review these fracture types assuming you have decided to treat them operatively and the patient is suitable for surgery. Please review the list of proximal humerus fracture types and add cases or make comments if you wish to do so. These suggestions will then be considered for the second round of this Delphi study. We have divided the list of cases in a list for Open Reduction Internal Fixation (ORIF) or Arthroplasty. We will start with the fracture types treated with ORIF. If you would like to add a fracture type or make a comment, you can do so on the next page.

1. Proximal humerus fracture without dislocation, intra-articular involvement, complete shaft translation, varus- or valgus malalignment, or neurovascular injury
2. Proximal humerus fracture with:

a. Dislocation of the humeral head to the axilla

b. “Locked” posterior fracture-dislocation of the humeral head (head fixated behind posterior glenoid with reversed Hill Sachs lesion)

c. Head-split fracture (≥35% intra-articular involvement)

d. (almost) Complete translation of shaft (<5% overlap between humeral head- and shaft)

e. Varus malalignment of the humeral head (neck-shaft angle ≤100 °)

f. Valgus malalignment of the humeral head (neck-shaft angle ≥150°)

3. Proximal humerus fracture with plexopathy
4. Proximal humerus fracture with vascular injury and active bleeding
5. Proximal humerus fracture with vascular compromise (e.g. intact distal vascularisation, pseudo-aneurysm)

List of proximal humerus fractures - ORIF - List of proximal humerus fractures - ORIF

Number	Question	Answers
	Please review the list of proximal humerus fracture types treated with ORIF carefully. We ask you to review these fracture types assuming you have decided to treat them operatively and the patient is suitable for surgery.	
1.1	List of proximal humerus fracture types treated with ORIF	
1.2	Do you want to comment on the provided list or add fracture types to the list?	◯ Yes<br>◯ No
1.2.1	***If 'Do you want to comment on the provided list or add fracture types to the list?' is equal to 'Yes' answer this question:***<br>Please provide your comment or the fracture type(s) you would like to add to the list.	

List of proximal humerus fractures - ORIF - Outro

Thank you for your insights. We will now present the list of proximal humerus fracture types treated with arthroplasty. This is the final part of the first round of this three-round survey study.

Survey 'List of Proximal Humerus Fractures - Arthroplasty'

List of Proximal Humerus Fractures - Arthroplasty - Introduction

Please review the list of proximal humerus fracture types treated with **arthroplasty** carefully. We ask you to review these fracture types assuming you have decided to treat them operatively and the patient is suitable for surgery. If you would like to add a fracture type or make a comment, you can do so on the next page.

1. Proximal humerus fracture without dislocation of the humeral head to the axilla or neurovascular injury
2. Proximal humerus fracture with dislocation of the humeral head to the axilla without plexopathy
3. Proximal humerus fracture with dislocation of the humeral head to the axilla and plexopathy (distal motor and/or sensory symptoms)
4. Proximal humerus fracture with axillary nerve injury and compromised deltoid function
5. Proximal humerus fracture with vascular compromise (e.g. intact distal vascularisation, pseudo-aneurysm)

List of Proximal Humerus Fractures - Arthroplasty - List of proximal humerus fractures - arthroplasty

Number	Question	Answers
	Please review the list of proximal humerus fracture types treated with arthroplasty carefully. We ask you to review these fracture types assuming you have decided to treat them operatively and the patient is suitable for surgery.	
1.1	List of proximal humerus fracture types treated with arthroplasty	
1.2	Do you want to comment on the provided list or add fracture types to the list?	◯ Yes<br>◯ No
1.2.1	***If 'Do you want to comment on the provided list or add fracture types to the list?' is equal to 'Yes' answer this question:***<br>Please provide your comment or the fracture type(s) you would like to add to the list.	

List of Proximal Humerus Fractures - Arthroplasty - Outro

Thank you for completing the final part of this first-round survey. This is the final opportunity to go back and review your previous answers. Once you complete the survey, it will be locked and your answers will be definitive.

Delphi Timing of Surgery After PHFs - Round 1 - Outro

Thank you for completing the first of three rounds in our Delphi study. We will evaluate your insights and reach out to you with the second survey in **six** weeks.
