# Supplementary material for: What is the preferred timing for surgery after proximal humerus fractures: an e-modified Delphi Survey Study
Source: JSES Rev Rep Tech. 2026 Aug 22;6(4):100865. doi: 10.1016/j.xrrt.2026.100865 (PMC13599625; doi:10.1016/j.xrrt.2026.100865)
Supplement: Supplementary file 2 [file mmc2.pdf]

Survey Package 'Delphi Timing of Surgery after PHFs - Round 2'

Survey 'Personal information round 2'

Personal information round 2 - Introduction

Dear panellist,

We want to thank you for the valuable insights and feedback we received from you in the first round. We noticed some confusion regarding the purpose of this study in some participants. We would like to reiterate that the primary objective of this study is to establish expert consensus on the **timing** of surgical intervention for *closed* and *isolated* proximal humerus fractures, rather than to evaluate the **indications** for surgery. We ask that you review the proposed fracture types under the assumption that a decision for operative treatment has already been made and that the patient is suitable for surgery. In light of your suggestions, we have added the proposed proximal humerus fracture patterns to the existing lists introduced in round 1.

In the second round of this e-modified Delphi study, we ask you for your opinion on the best timing for the fracture types listed in the first round, including the newly added ones. We ask you to share justifications for your answers to gain insight into factors surgeons consider when deciding on the best timing of surgical management.

The survey will start with a few questions regarding your personal information. This information is used to track participation across rounds, allowing us to analyse consensus stability.

You will then be asked to indicate within how many days you would perform surgery for each listed fracture type. The fracture types are divided into two categories: ORIF and arthroplasty.

Based on literature (up to June 2025) and clinical experience, we have defined the following timeframes for surgery:

• Within 2 days of the initial trauma

• Within 3 to 5 days of the initial trauma

• Within 6 to 14 days of the initial trauma

• After 14 days of the initial trauma

Personal information round 2 - Personal information

Number	Question	Answers
	All information will remain confidential and all answers are anonymous.	
1.1	Please state your first name.	
1.2	Please state your initials.	

1.3	Please state your surname.	
1.4	Do you have any conflicts of interest to declare	◯ Yes<br>◯ No
1.4.1	***If 'Do you have any conflicts of interest to declare' is equal to 'Yes' answer this question:***<br>Please state your conflict of interest.	

Survey 'Timing surgery round 2 - ORIF'

Timing surgery round 2 - ORIF - Introduction

In the following survey, you will decide within how many days you would perform surgery on the given fracture types with optional justification of your choice. Please note that this study attempts to establish consensus on the **timing** of surgery, not indications for surgery. We will start with ORIF.

Timing surgery round 2 - ORIF - Timing of surgery round 2 - ORIF

Number	Question	Answers
	Assuming you perform an ORIF, within how many days since the injury would you perform this surgery in the following cases?	
1.1	Proximal humerus fracture without the following characteristics: dislocation, intra-articular involvement, complete shaft translation, varus- or valgus malalignment, tuberosity displacement, unstable medial hinge, or neurovascular injury	◯ Within two days of the initial trauma (10.2%)<br>◯ Within three to five days of the initial trauma (38.8%; see rationale)<br>◯ Within six to fourteen days of the initial trauma (42.9%; see rationale)<br>◯ After fourteen days of the initial trauma (8.1%)
1.2	Please elaborate on your choice of timing if you wish to do so	
1.3	Proximal humerus fracture with dislocation of the humeral head to the axilla	◯ Within two days of the initial trauma (10.2%)<br>◯ Within three to five days of the initial trauma (38.8%; see rationale)<br>◯ Within six to fourteen days of the initial trauma (42.9%; see rationale)<br>◯ After fourteen days of the initial trauma (8.1%)
1.4	Please elaborate on your choice of timing if you wish to do so	

1.5	Proximal humerus fracture with “locked” posterior fracture-dislocation of the humeral head (head fixated behind posterior glenoid with reversed Hill Sachs lesion)	◯ Within two days of the initial trauma (10.2%)<br>◯ Within three to five days of the initial trauma (38.8%; see rationale)<br>◯ Within six to fourteen days of the initial trauma (42.9%; see rationale)<br>◯ After fourteen days of the initial trauma (8.1%)
1.6	Please elaborate on your choice of timing if you wish to do so	
1.7	Proximal humerus fracture with ≥35% intra-articular involvement (formerly known as 'head-split')	◯ Within two days of the initial trauma (10.2%)<br>◯ Within three to five days of the initial trauma (38.8%; see rationale)<br>◯ Within six to fourteen days of the initial trauma (42.9%; see rationale)<br>◯ After fourteen days of the initial trauma (8.1%)
1.8	Please elaborate on your choice of timing if you wish to do so	
1.9	(almost) Complete translation of shaft (<5% overlap between humeral head- and shaft)	◯ Within two days of the initial trauma (10.2%)<br>◯ Within three to five days of the initial trauma (38.8%; see rationale)<br>◯ Within six to fourteen days of the initial trauma (42.9%; see rationale)<br>◯ After fourteen days of the initial trauma (8.1%)
1.10	Please elaborate on your choice of timing if you wish to do so	
1.11	Varus malalignment of the humeral head (neck-shaft angle ≤100 °)	◯ Within two days of the initial trauma (10.2%)<br>◯ Within three to five days of the initial trauma (38.8%; see rationale)<br>◯ Within six to fourteen days of the initial trauma (42.9%; see rationale)<br>◯ After fourteen days of the initial trauma (8.1%)

1.12	Please elaborate on your choice of timing if you wish to do so	
1.13	Valgus malalignment of the humeral head (neck-shaft angle ≥150°)	◯ Within two days of the initial trauma (10.2%)<br>◯ Within three to five days of the initial trauma (38.8%; see rationale)<br>◯ Within six to fourteen days of the initial trauma (42.9%; see rationale)<br>◯ After fourteen days of the initial trauma (8.1%)
1.14	Please elaborate on your choice of timing if you wish to do so	
1.15	Tuberosity (greater and/or lesser) displacement > 5 mm	◯ Within two days of the initial trauma (10.2%)<br>◯ Within three to five days of the initial trauma (38.8%; see rationale)<br>◯ Within six to fourteen days of the initial trauma (42.9%; see rationale)<br>◯ After fourteen days of the initial trauma (8.1%)
1.16	Please elaborate on your choice of timing if you wish to do so	
1.17	Unstable disruption of the medial hinge	◯ Within two days of the initial trauma (10.2%)<br>◯ Within three to five days of the initial trauma (38.8%; see rationale)<br>◯ Within six to fourteen days of the initial trauma (42.9%; see rationale)<br>◯ After fourteen days of the initial trauma (8.1%)
1.18	Please elaborate on your choice of timing if you wish to do so	

1.19	Proximal humerus fracture with plexopathy with distal motor and/or sensory symptoms (directly caused by fracture)	◯ Within two days of the initial trauma (10.2%)<br>◯ Within three to five days of the initial trauma (38.8%; see rationale)<br>◯ Within six to fourteen days of the initial trauma (42.9%; see rationale)<br>◯ After fourteen days of the initial trauma (8.1%)
1.20	Please elaborate on your choice of timing if you wish to do so	
1.21	Proximal humerus fracture with vascular injury and active bleeding	◯ Within two days of the initial trauma (10.2%)<br>◯ Within three to five days of the initial trauma (38.8%; see rationale)<br>◯ Within six to fourteen days of the initial trauma (42.9%; see rationale)<br>◯ After fourteen days of the initial trauma (8.1%)
1.22	Please elaborate on your choice of timing if you wish to do so	
1.23	Proximal humerus fracture with vascular compromise (e.g. intact distal vascularisation, pseudo-aneurysm)	◯ Within two days of the initial trauma (10.2%)<br>◯ Within three to five days of the initial trauma (38.8%; see rationale)<br>◯ Within six to fourteen days of the initial trauma (42.9%; see rationale)<br>◯ After fourteen days of the initial trauma (8.1%)
1.24	Please elaborate on your choice of timing if you wish to do so	

Timing surgery round 2 - ORIF - Outro

Thank you for your insights. We will now proceed with arthroplasty.

Survey 'Timing surgery round 2 - Arthroplasty'

Timing surgery round 2 - Arthroplasty - Timing surgery round 2 - Arthroplasty

Number	Question	Answers
	Assuming you perform an arthroplasty, within how many days since the injury, would you perform this surgery in the following cases?	
1.1	Proximal humerus fracture without dislocation of the humeral head to the axilla or neurovascular injury	◯ Within two days of the initial trauma (10.2%)<br>◯ Within three to five days of the initial trauma (38.8%; see rationale)<br>◯ Within six to fourteen days of the initial trauma (42.9%; see rationale)<br>◯ After fourteen days of the initial trauma (8.1%)
1.2	Please elaborate on your choice of timing if you wish to do so	
1.3	Proximal humerus fracture with dislocation of the humeral head to the axilla without plexopathy	◯ Within two days of the initial trauma (10.2%)<br>◯ Within three to five days of the initial trauma (38.8%; see rationale)<br>◯ Within six to fourteen days of the initial trauma (42.9%; see rationale)<br>◯ After fourteen days of the initial trauma (8.1%)
1.4	Please elaborate on your choice of timing if you wish to do so	
1.5	Proximal humerus fracture with dislocation of the humeral head to the axilla and plexopathy (distal motor and/or sensory symptoms)	◯ Within two days of the initial trauma (10.2%)<br>◯ Within three to five days of the initial trauma (38.8%; see rationale)<br>◯ Within six to fourteen days of the initial trauma (42.9%; see rationale)<br>◯ After fourteen days of the initial trauma (8.1%)

1.6	Please elaborate on your choice of timing if you wish to do so	
1.7	Proximal humerus fracture with axillary nerve injury and compromised deltoid function	◯ Within two days of the initial trauma<br>◯ Within three to five days of the initial trauma<br>◯ Within six to fourteen days of the initial trauma<br>◯ After fourteen days of the initial trauma<br>◯ No operation
1.8	Please elaborate on your choice of timing if you wish to do so	
1.9	Proximal humerus fracture with vascular compromise (e.g. intact distal vascularisation, pseudo-aneurysm)	◯ Within two days of the initial trauma (10.2%)<br>◯ Within three to five days of the initial trauma (38.8%; see rationale)<br>◯ Within six to fourteen days of the initial trauma (42.9%; see rationale)<br>◯ After fourteen days of the initial trauma (8.1%)
1.10	Please elaborate on your choice of timing if you wish to do so	
1.11	Proximal humerus fracture with “locked” posterior fracture-dislocation of the humeral head (head fixated behind posterior glenoid with reversed Hill Sachs lesion)	◯ Within two days of the initial trauma (10.2%)<br>◯ Within three to five days of the initial trauma (38.8%; see rationale)<br>◯ Within six to fourteen days of the initial trauma (42.9%; see rationale)<br>◯ After fourteen days of the initial trauma (8.1%)
1.12	Please elaborate on your choice of timing if you wish to do so	

1.13	Proximal humerus fracture with ≥35% intra-articular involvement (formerly known as 'head-split')	◯ Within two days of the initial trauma (10.2%)<br>◯ Within three to five days of the initial trauma (38.8%; see rationale)<br>◯ Within six to fourteen days of the initial trauma (42.9%; see rationale)<br>◯ After fourteen days of the initial trauma (8.1%)
1.14	Please elaborate on your choice of timing if you wish to do so	
1.15	Varus malalignment of the humeral head (neck-shaft angle ≤100 °)	◯ Within two days of the initial trauma (10.2%)<br>◯ Within three to five days of the initial trauma (38.8%; see rationale)<br>◯ Within six to fourteen days of the initial trauma (42.9%; see rationale)<br>◯ After fourteen days of the initial trauma (8.1%)
1.16	Please elaborate on your choice of timing if you wish to do so	
1.17	(almost) Complete translation of shaft (<5% overlap between humeral head- and shaft)	◯ Within two days of the initial trauma (10.2%)<br>◯ Within three to five days of the initial trauma (38.8%; see rationale)<br>◯ Within six to fourteen days of the initial trauma (42.9%; see rationale)<br>◯ After fourteen days of the initial trauma (8.1%)
1.18	Please elaborate on your choice of timing if you wish to do so	

Timing surgery round 2 - Arthroplasty - Outro

Thank you for completing the final part of this second round survey!

Delphi Timing of Surgery after PHFs - Round 2 - Outro

Thank you for completing the second round survey for this Delphi study. This is the final chance to go back and review your answers if you wish to do so. Once you complete the survey, it will be locked and your answers will be definitive. We will reach out to you in **six** weeks with the final survey.
