# Supplementary material for: What is the preferred timing for surgery after proximal humerus fractures: an e-modified Delphi Survey Study
Source: JSES Rev Rep Tech. 2026 Aug 22;6(4):100865. doi: 10.1016/j.xrrt.2026.100865 (PMC13599625; doi:10.1016/j.xrrt.2026.100865)
Supplement: Supplementary file 3 [file mmc3.pdf]

Survey Package 'Delphi Timing of Surgery after PHFs - Round 3'

Delphi Timing of Surgery after PHFs - Round 3 - Introduction

Dear panellist,

We want to thank you again for your participation and insights in Round 2. Our study aims to to establish expert consensus on the **timing** of surgical intervention for proximal humerus fractures. We ask that you review the proposed fracture types under the assumption that a decision for operative treatment has already been made and that the patient is suitable for surgery. We have summarised the results and the given rationales from Round 2 to aid your decision-making.

In the third and final round of this e-modified Delphi study, you will have the opportunity to re-assess the timing for each fracture type using the same timeframes, while considering the summary of responses from Round 2. We have reported the percentages for each timeframe with the corresponding rationales. Please complete this survey even if you have not submitted a response previously.

The survey will start with a few questions regarding your personal information. This information is used to track participation across rounds, allowing us to analyse consensus stability.

You will then be asked again to indicate within how many days you would perform surgery for each listed fracture type (first ORIF, then arthroplasty), while considering the summary of the previous round.

Survey 'Personal information round 3'

Personal information round 3 - Personal information

Number	Question	Answers
	All information will remain confidential and all answers are anonymous.	
1.1	Please state your first name.	
1.2	Please state your initials.	
1.3	Please state your surname.	

1.4	Do you have any conflicts of interest to declare	◯ Yes<br>◯ No
1.4.1	***If 'Do you have any conflicts of interest to declare' is equal to 'Yes' answer this question:***<br>Please state your conflict of interest.	

Survey 'Timing surgery round 3 - ORIF'

Timing surgery round 3 - ORIF - Introduction

In the following survey, you will have the opportunity to re-assess your answers from Round 2. We have summarised the results from the previous round for each fracture type. The percentages are displayed within parentheses after the corresponding timeframe and we have indicated if rationale is available. The summarised **rationales** are available via the **'help-text'** button next to the question. Our study aims to to establish expert consensus on the *timing* of surgical intervention for proximal humerus fractures.

Timing surgery round 3 - ORIF - Timing of surgery round 3 - ORIF

Number	Question	Answers
	Assuming you perform an ORIF, within how many days since the injury would you perform this surgery in the following cases, while considering the summarised results? The percentages are listed within parentheses after each timeframe and the rationales are available via the help button next to the question.	
1.1	Proximal humerus fracture without the following characteristics: dislocation, intra-articular involvement, complete shaft translation, varus- or valgus malalignment, tuberosity displacement, unstable medial hinge, or neurovascular injury	○ Within two days of the initial trauma (10.2%)<br>○ Within three to five days of the initial trauma (38.8%; see rationale)<br>○ Within six to fourteen days of the initial trauma (42.9%; see rationale)<br>○ After fourteen days of the initial trauma (8.1%)
1.2	Proximal humerus fracture with dislocation of the humeral head to the axilla	○ Within two days of the initial trauma (85.8%; see rationale)<br>○ Within three to five days of the initial trauma (12.2%)<br>○ Within six to fourteen days of the initial trauma (0%)<br>○ After fourteen days of the initial trauma (2.0%)
1.3	Proximal humerus fracture with “locked” posterior fracture-dislocation of the humeral head (head fixated behind posterior glenoid with reversed Hill Sachs lesion)	○ Within two days of the initial trauma (61.3%; see rationale)<br>○ Within three to five days of the initial trauma (30.6%; see rationale)<br>○ Within six to fourteen days of the initial trauma (6.1%)<br>○ After fourteen days of the initial trauma (2.0%)

1.4	Proximal humerus fracture with ≥35% intra-articular involvement (formerly known as 'head-split')	◯ Within two days of the initial trauma (24.5%; see rationale)<br>◯ Within three to five days of the initial trauma (53.1%; see rationale)<br>◯ Within six to fourteen days of the initial trauma (16.3%)<br>◯ After fourteen days of the initial trauma (6.1%)
1.5	(almost) Complete translation of shaft (<5% overlap between humeral head- and shaft)	◯ Within two days of the initial trauma (20.4%; see rationale)<br>◯ Within three to five days of the initial trauma (40.8%; see rationale)<br>◯ Within six to fourteen days of the initial trauma (34.7%; see rationale)<br>◯ After fourteen days of the initial trauma (4.1%)
1.6	Varus malalignment of the humeral head (neck-shaft angle ≤100 °)	◯ Within two days of the initial trauma (6.1%)<br>◯ Within three to five days of the initial trauma (44.9%; see rationale)<br>◯ Within six to fourteen days of the initial trauma (42.9%; see rationale)<br>◯ After fourteen days of the initial trauma (6.1%; see rationale)
1.7	Valgus malalignment of the humeral head (neck-shaft angle ≥150°)	◯ Within two days of the initial trauma (4.1%)<br>◯ Within three to five days of the initial trauma (44.9%; see rationale)<br>◯ Within six to fourteen days of the initial trauma (42.9%; see rationale)<br>◯ After fourteen days of the initial trauma (6.1%; see rationale)
1.8	Tuberosity (greater and/or lesser) displacement > 5 mm	◯ Within two days of the initial trauma (4.1%)<br>◯ Within three to five days of the initial trauma (40.8%; see rationale)<br>◯ Within six to fourteen days of the initial trauma (49.0%; see rationale)<br>◯ After fourteen days of the initial trauma (6.1%; see rationale)
1.9	Unstable disruption of the medial hinge	◯ Within two days of the initial trauma (18.4%)<br>◯ Within three to five days of the initial trauma (44.9%; see rationale)<br>◯ Within six to fourteen days of the initial trauma (34.7%; see rationale)<br>◯ After fourteen days of the initial trauma (2.0%; see rationale)

1.10	Proximal humerus fracture with plexopathy with distal motor and/or sensory symptoms (directly caused by fracture)	◯ Within two days of the initial trauma (77.6%; see rationale)<br>◯ Within three to five days of the initial trauma (18.4%; see rationale)<br>◯ Within six to fourteen days of the initial trauma (2.0%)<br>◯ After fourteen days of the initial trauma (2.0%)
1.11	Proximal humerus fracture with vascular injury and active bleeding	◯ Within two days of the initial trauma (100%; see rationale)<br>◯ Within three to five days of the initial trauma (0%)<br>◯ Within six to fourteen days of the initial trauma (0%)<br>◯ After fourteen days of the initial trauma (0%)
1.12	Proximal humerus fracture with vascular compromise (e.g. intact distal vascularisation, pseudo-aneurysm)	◯ Within two days of the initial trauma (91.9%; see rationale)<br>◯ Within three to five days of the initial trauma (2.0%)<br>◯ Within six to fourteen days of the initial trauma (4.1%; see rationale)<br>◯ After fourteen days of the initial trauma (2.0%)

Survey 'Timing surgery round 3 - Arthroplasty'

Timing surgery round 3 - Arthroplasty - Introduction

Thank you for your insights. We will now proceed with arthroplasty. Again, the summaries are listed after each corresponding timeframe and summarised **rationales** are available via the **'help-text'** button next to the questions.

Timing surgery round 3 - Arthroplasty - Timing surgery round 3 - Arthroplasty

Number	Question	Answers
	Assuming you perform an arthroplasty, within how many days since the injury, would you perform this surgery in the following cases, while considering the summarised results? The percentages are listed within parentheses after each timeframe and the rationales are available via the help button next to the question.	
1.1	Proximal humerus fracture without dislocation of the humeral head to the axilla or neurovascular injury	◯ Within two days of the initial trauma (6.1%)<br>◯ Within three to five days of the initial trauma (22.4%; see rationale)<br>◯ Within six to fourteen days of the initial trauma (57.2%; see rationale)<br>◯ After fourteen days of the initial trauma (14.3%)
1.2	Proximal humerus fracture with dislocation of the humeral head to the axilla without plexopathy	◯ Within two days of the initial trauma (24.5%; see rationale)<br>◯ Within three to five days of the initial trauma (47.0%; see rationale)<br>◯ Within six to fourteen days of the initial trauma (22.4%; see rationale)<br>◯ After fourteen days of the initial trauma (6.1%)
1.3	Proximal humerus fracture with dislocation of the humeral head to the axilla and plexopathy (distal motor and/or sensory symptoms)	◯ Within two days of the initial trauma (79.7%; see rationale)<br>◯ Within three to five days of the initial trauma (16.3%; see rationale)<br>◯ Within six to fourteen days of the initial trauma (2.0)<br>◯ After fourteen days of the initial trauma (2.0%)

1.4	Proximal humerus fracture with axillary nerve injury and compromised deltoid function	○ Within two days of the initial trauma (44.9%; see rationale)<br>○ Within three to five days of the initial trauma (18.3%; see rationale)<br>○ Within six to fourteen days of the initial trauma (8.2%)<br>○ After fourteen days of the initial trauma (8.2%)<br>○ No operation (20.4%; see rationale)
1.5	Proximal humerus fracture with vascular compromise (e.g. intact distal vascularisation, pseudo-aneurysm)	○ Within two days of the initial trauma (83.7%; see rationale)<br>○ Within three to five days of the initial trauma (12.2%; see rationale)<br>○ Within six to fourteen days of the initial trauma (0%)<br>○ After fourteen days of the initial trauma (4.1%)
1.6	Proximal humerus fracture with “locked” posterior fracture-dislocation of the humeral head (head fixated behind posterior glenoid with reversed Hill Sachs lesion)	○ Within two days of the initial trauma (24.5%; see rationale)<br>○ Within three to five days of the initial trauma (40.8%; see rationale)<br>○ Within six to fourteen days of the initial trauma (28.6%; see rationale)<br>○ After fourteen days of the initial trauma (6.1%)
1.7	Proximal humerus fracture with ≥35% intra-articular involvement (formerly known as 'head-split')	○ Within two days of the initial trauma (4.1%)<br>○ Within three to five days of the initial trauma (22.4%; see rationale)<br>○ Within six to fourteen days of the initial trauma (61.3%; see rationale)<br>○ After fourteen days of the initial trauma (12.2%)
1.8	Varus malalignment of the humeral head (neck-shaft angle ≤100 °)	○ Within two days of the initial trauma (2.0%)<br>○ Within three to five days of the initial trauma (24.5%; see rationale)<br>○ Within six to fourteen days of the initial trauma (59.2%; see rationale)<br>○ After fourteen days of the initial trauma (14.3%)

1.9	(almost) Complete translation of shaft (<5% overlap between humeral head- and shaft)	◯ Within two days of the initial trauma (2.0%)<br>◯ Within three to five days of the initial trauma (32.7%; see rationale)<br>◯ Within six to fourteen days of the initial trauma (55.1%; see rationale)<br>◯ After fourteen days of the initial trauma (10.2%)

Delphi Timing of Surgery after PHFs - Round 3 - Outro

Thank you so much for completing the third round and final survey for this Delphi study! This is the final chance to go back and review your answers if you wish to do so. Once you complete the survey, it will be locked and your answers will be definitive. We will analyse and write up the results in the coming weeks. All participants who completed the three rounds will receive group authorship.
